# Effect of assertive case management intervention on suicide attempters with comorbid Axis I and II psychiatric diagnoses: secondary analysis of a randomised controlled trial

**DOI:** 10.1186/s12888-020-02723-9

**Published:** 2020-06-16

**Authors:** Kazunobu Norimoto, Katsumi Ikeshita, Toshifumi Kishimoto, Kazuo Okuchi, Naohiro Yonemoto, Tatsuya Sugimoto, Fuminori Chida, Shigero Shimoda, Yoshio Hirayasu, Chiaki Kawanishi

**Affiliations:** 1 Department of Psychiatry, Nara Medical University, 840 Shijo-Cho Kashihara, Nara, 634-8521 Japan; 2 Department of Emergency and Critical Care Medicine, Nara Medical University, 840 Shijo-Cho Kashihara, Nara, 634-8521 Japan; 3 ICHI Mental Clinic Nipponbashi, 1-3-1 Nipponbashi, Chuo-ku, Osaka, 542-0073 Japan; 4 Department of Neuropsychopharmacology, National Center of Neurology and Psychiatry, 4-1-1 Ogawa-Higasi, Kodaira, Tokyo, 187-8551 Japan; 5 Department of Public Health, Juntendo University School of Medicine, 2-1-1 Hongo, Bunkyo-ku, Tokyo, 113-8421 Japan; 6 Department of Psycho-oncology, Shizuoka Cancer Center, 1007 Shimonagakubo, Nagaizumi-cho, Sunto-gun, Shizuoka Prefecture, Nagaizumi, 411-8777 Japan; 7 Department of Neuropsychiatry, Iwate Medical University, 1-1-1 Idaidori, Yahaba-cho, Shiwa-gun, Iwate-Prefecture, 028-3694 Japan; 8 Hirayasu Hospital, 346 Kyozuka, Urasoe, Okinawa, 901-2111 Japan; 9 Department of Neuropsychiatry, Sapporo Medical University Graduate School of Medicine, 291 S-1, W-16, Chuo-ku, Sapporo, 060-8543 Japan

**Keywords:** Suicide, Suicide attempt, Assertive case management intervention, Comorbidity, Axis I, Axis II

## Abstract

**Background:**

Most suicide attempters suffer from psychiatric disorders, which are often comorbid with personality disorders. The effects of intervention on patients who have attempted suicide with comorbid Axis I and II diagnoses have not been fully elucidated. We evaluated whether assertive case management can reduce the repetition of suicidal behaviours in patients who had attempted suicide with comorbid Axis I and II diagnoses.

**Methods:**

This study was a secondary analysis of a randomised controlled trial investigating whether assertive case management could reduce the repetition of suicide attempts, compared with enhanced usual care. Subjects were divided into those who had comorbid Axis I and II diagnoses (Axis I + II group), and those who had an Axis I diagnosis without Axis II comorbidity (Axis I group). Outcome measures were compared between patients receiving a case management intervention and patients receiving enhanced usual care, as allocated. The primary outcome measure was the incidence proportion of the first episode of recurrent suicidal behaviour at 6 months after randomisation. We calculated risk ratios (RR) with 95% confidence intervals (CI) at 6 months and 12 months after randomisation of patients in the Axis I and Axis I + II groups.

**Results:**

Of 914 enrolled patients, 120 (13.1%) were in the Axis I + II group, and 794 (86.9%) were in the Axis I group. Assertive case management was significantly effective for the Axis I group on the primary outcome at 6 months (risk ratio [RR] 0.51, 95% confidence intervals [CI] 0.31 to 0.84). The RR of the Axis I + II group was 0.44 (95% CI 0.14 to 1.40).

**Conclusions:**

Assertive case management not only had an effect on patients who had attempted suicide with only Axis I disorders but may also have a similar effect on patients with comorbid Axis I and II disorders.

## Background

Attempted suicide is an increasingly prevalent public health concern internationally [1]. Most suicide attempters suffer from psychiatric disorders, and their psychiatric disorders are often comorbid with Diagnostic and Statistical Manual of Mental Disorders 4th Edition (DSM-IV)-Axis II conditions, including personality disorders. Of patients who attempted suicide, 44% were reported to have comorbid personality disorders [2]. In addition, 66% of patients with a personality disorder admitted to an emergency department for attempted suicide were reported to have a concomitant Axis I disorder [3]. Moreover, the risk of suicide in patients with Axis I-Axis II comorbidity was reported to be higher than that in those with Axis I disorders only [4]. Patients with comorbid psychiatric and personality disorders repeated suicide attempts more often than patients without both of these disorders [2].

Several reviews have examined the effects of interventions on preventing suicide attempts [5–7]. However, the effects of intervention on suicide-attempting patients with comorbid Axis I and II psychiatric diagnoses have not been fully elucidated.

We recently examined the effects of assertive case management on repeat suicide attempts in the emergency department setting (the ACTION- J study). The intervention, which lasted for at least 18 months, was introduced by case managers during emergency department admissions for suicide attempts. It was based on psychiatric diagnoses, social risks, and patient demands. The participants had received a primary diagnosis of Axis I psychiatric disorder. Compared with usual care, the intervention significantly reduced the number of individuals with first recurrent suicide attempts for up to 6 months. The intervention of ACTION-J have already been published in detail [8, 9].

### Objectives

Our aim was to evaluate whether assertive case management can reduce the repetition of suicidal behaviours in patients who had attempted suicide with Axis I-Axis II comorbidity, compared with patients receiving enhanced usual care.

## Methods

### Participant recruitment and selection

**Fig. 1:**
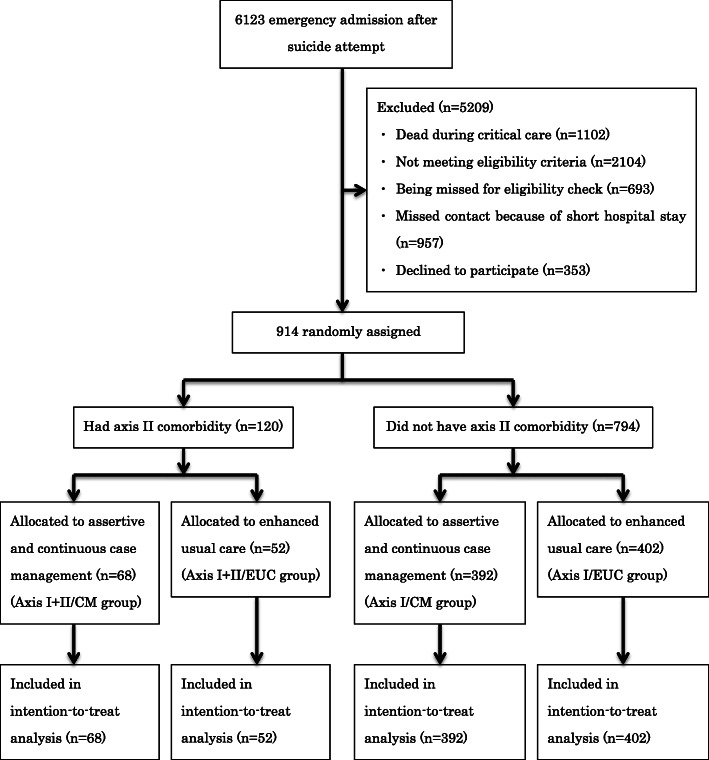
Trial profile

This study involved a secondary analysis of data from the ACTION-J trial. Figure 1 shows a flow chart of the recruitment process. This was a multi-centre randomised controlled trial involving 17 Japanese hospitals with both an emergency department and a psychiatric department [8, 9]. Participants in ACTION-J were adult patients (aged 20 years and older) admitted to the emergency department because of a suicide attempt, who received a primary diagnosis of a DSM-IV Axis I disorder. Participants were restricted to primary diagnosis of an Axis I disorder because the case management intervention was developed for those patients. The diagnosis was based on a structured interview with the Mini-International Neuropsychiatric Interview (MINI) to provide psychiatric diagnosis, including Axis II disorders. Axis I and Axis II psychiatric diagnoses were made in accord with the DSM-IV Text Revision (DSM-IV-TR). Suicidal intent at the indexed suicide attempt episode was confirmed using the Suicide Intent Scale. The inclusion and exclusion criteria have been reported elsewhere [8, 9].

All participants provided written informed consent prior to study enrolment. The study protocol was approved by the Central Research Ethics Committee of the study sponsor (Japan Foundation for Neuroscience and Mental Health, Tokyo, Japan) and by the local ethics committees of all participating hospitals.

### Procedures

If a physician in an emergency facility suspects that a patient has made a suicide attempt, the physician will contact a psychiatrist. The psychiatrist will collect information and make a psychiatric diagnosis upon examination of the patient. Participants also received a psychoeducation session in the emergency department before randomisation. Participants who were randomly assigned to the control group received enhanced usual care at the participating emergency departments. After randomisation, case managers gave these participants an information pamphlet listing available social resources (health care-based and local government services) each time they visited for periodic assessments (6 months and 18 months after randomisation, then annually until the end of the study) [9].

The ACTION-J intervention consisted of 1) Periodic contact (either face-to-face or by telephone) with participants during their stay in the emergency department and after discharge, 2) Collection of information about each participant’s treatment status and social problems that might negatively affect their treatment adherence, 3) Encouragement of participants to adhere to psychiatric treatment, 4) Coordination of appointments with psychiatrists and primary care physicians, 5) Encouragement of participants who discontinued psychiatric treatment to return to treatment, 6) Referrals to social services and private support organisations, and coordination for use of these resources to accommodate the individual needs of patients, 7) Provision of psychoeducation content and information about social resources through a dedicated website. The ACTION-J intervention was provided by case managers who were trained mental health experts (psychiatrists, nurses, social workers, or clinical psychologists). The case managers periodically contacted participants assigned to the intervention group for 18 months following randomisation (at week 1 and at months 1, 2, 3, 6, 12, and 18) during their stay in the emergency department and after discharge. When applicable, they contacted the participants every 6 months until the end of the trial (June 30, 2011). The details of the study procedures have been reported elsewhere [8, 9].

### Outcomes

The primary outcome measure was the incidence proportion of the first episode of recurrent suicidal behaviour (attempted suicide or dying by suicide) at 6 months after randomisation. Because recurrent suicidal behaviour of borderline personality disorder (BPD) showed a steeper decline with mentalisation-based treatment compared with structured clinical management [10], cognitive therapy for the prevention of suicide induced rapid changes within 6 months on negative problem orientation and impulsivity/carelessness problem-solving style [11]. In addition, the incidence proportion of recurrent suicidal behaviour in the intervention group significantly decreased until 6 months after randomisation compared with the control group in the ACTION-J [8]. Suicide attempts were defined as self-poisoning (overdose) or self-injury carried out with apparent suicidal intent [12].

The secondary outcome measure was the incidence proportion of the first episode of recurrent suicidal behaviour at 12 months after randomisation. It has been reported that the effect of intervention for recurrent suicidal behaviour of BPD emerges gradually [13], and active contact and follow-up type interventions can reduce the likelihood of repetition of suicide attempts within 12 months [5].

Outcome assessors who were masked to group assignment collected information about suicide attempts from participants or their family members via direct interviews. Evaluation of outcomes was performed in face-to-face or telephone interviews 6 and 18 months after randomisation, then annually until the end of the study. An event review committee independently assessed all events related to the study outcomes.

### Analysis

We divided the subjects into a group with Axis II comorbidity (Axis I + II group) and a group without Axis II comorbidity (Axis I group). Each group included participants who received a case management intervention or enhanced usual care. Thus, we divided each group into two based on whether participants received case management intervention or enhanced usual care (Case management group; CM group, Enhanced usual care group; EUC group, respectively). We constructed and compared the four groups as follows: Axis I + II/CM group vs Axis I/CM group, and Axis I + II/EUC group vs Axis I/EUC group.

We calculated risk ratios (RR) with 95% confidence intervals (CI) at 6 months and 12 months after randomisation of the Axis I group and Axis I + II group. In addition, we described an overall survival curve using the Kaplan-Meier method. We also made adjustments using regression models with the following randomisation factors: gender (male vs female), age (< 40 vs ≥ 40 years), and history of previous suicide attempts before the current episode (yes vs no). Selection of the age category was based on stratification of randomisation in the ACTION-J trial and a previous clinical study of an emergency department in Japan. Risk was reported to be significantly different depending on age (< 40 years and > 40 years). Linearity of the risk was not confirmed in the report [3]. All analyses were based on the intention-to-treat principle and were explanatory in nature. Measuring RR provided helpful information for the analysis, but was not based on *p*-values. Specifically, the comparison of the Axis I group had a medium sample size and insufficient statistical power. For sensitivity analyses, we performed multiple imputations for missing data and used regression models to adjust for the randomisation factors [14]. Statistical analyses were performed using JMP 10.0.2 (SAS Institute, Inc., Cary, NC, USA) and SAS 9.4 (SAS Institute, Inc., Cary, NC, USA).

## Results

A total of 914 patients were enrolled in the study. Of these, 120 cases (13.1%) were patients with Axis II comorbidity (Axis I + II group), and, of these, 68 were randomly assigned to the assertive case management group (Axis I + II/CM group), while 52 were assigned to the enhanced usual care group (Axis I + II/EUC group). Meanwhile, 794 patients (86.9%) did not have Axis II comorbidity (Axis I group), and 392 of them were randomly assigned to the assertive case management group (Axis I/CM group) while 402 were assigned to enhanced usual care group (Axis I/EUC group) (Fig. 1). Baseline characteristics are shown in Table 1. The proportion of women was 73.5 in the Axis I + II/CM group and 67.3 in the Axis I + II/EUC group. The mean age was 33.9 and 33.0 years, respectively. The proportion of those who had made one or more suicide attempts was 76.5 and 76.9, respectively. The proportion of women in the Axis I/CM group was 54.3, and the proportion of women in the Axis I/EUC group was 53.7. The average age was 44.6 years and 42.8 years, respectively. The proportion of those who experienced one or more suicide attempts was 45.7 and 44.5, respectively.

**Table 1:** Baseline characteristics

	Axis I + II group		Axis I group	
	Intervention (*n* = 68) N (%)	Control (*n* = 52) N (%)	Intervention (*n* = 392) N (%)	Control (*n* = 402) N (%)
**Sex male/female**	18(26.5) /50(73.5)	17(32.7) /35(67.3)	179(45.7) /213(54.3)	186(46.3) /216(53.7)
**Age, mean (SD)**	33.9 (9.4)	33.0 (10.3)	44.6 (14.8)	42.8 (15.3)
**Primary psychiatric diagnosis**				
Substance-related disorder	6 (8.8)	6 (11.5)	13 (3.3)	20 (5.0)
Schizophrenia or other psychotic disorder	6 (8.8)	3 (5.8)	87 (22.2)	83 (20.6)
Mood disorder	31 (45.6)	27 (51.9)	184 (46.9)	184 (45.8)
Adjustment disorder	18 (26.5)	9 (17.3)	82 (20.9)	82 (20.4)
Other	7 (10.3)	7 (13.4)	26 (6.6)	33 (8.2)
**Comorbid Axis II diagnosis**				
Personality disorder	58 (85.3)	45 (86.5)		
Mental retardation	10 (14.7)	7 (13.5)		
**Visited a psychiatrist within 1 month before the suicide attempt**	51 (75.0)	41 (78.8)	209 (53.3)	216 (53.7)
**Education**				
Less than high school	22 (32.4)	20 (38.5)	93 (23.7)	88 (21.9)
High school	25 (36.8)	25 (48.1)	204 (52.0)	212 (52.7)
Beyond high school	21 (30.9)	7 (13.5)	95 (24.2)	102 (25.4)
**Employment status**				
Employed	31 (46.3)	17 (32.7)	163 (41.6)	189 (47.0)
Unemployed	35 (52.2)	34 (65.4)	208 (53.1)	186 (46.3)
Retired	0 (0.0)	1 (1.9)	11 (2.8)	15 (3.7)
Student	1 (1.5)	0 (0.0)	10 (2.6)	12 (3.0)
**Marital status**				
Married	15 (22.1)	15 (28.8)	165 (42.1)	180 (44.8)
Single	40 (58.8)	28 (53.8)	129 (32.9)	155 (38.6)
Divorced	13 (19.1)	9 (17.3)	81 (20.7)	52 (12.9)
Widowed	0 (0.0)	0 (0.0)	17 (4.3)	15 (3.7)
**Lives with partner or family**	17 (25.0)	9 (17.3)	96 (24.5)	75 (18.7)
**Previous suicide attempts**				
None	16 (23.5)	12 (23.1)	213 (54.3)	223 (55.5)
One or more times	52 (76.5)	40 (76.9)	179 (45.7)	179 (44.5)
**Method of the present suicide attempt**^a^				
Drug overdose	55 (80.9)	36 (69.2)	271 (69.1)	286 (71.1)
Gas	1 (1.5)	3 (5.8)	30 (7.7)	25 (6.2)
Laceration	9 (13.2)	7 (13.5)	67 (17.1)	64 (15.9)
Jumping from a high place	0 (0.0)	1 (1.9)	10 (2.6)	6 (1.5)
Intentional traffic-related injury	10 (14.7)	3 (5.8)	45 (11.4)	57 (14.2)
Hanging	1 (1.5)	6 (11.5)	26 (6.6)	20 (5.0)
Other	4 (5.9)	3 (5.8)	17 (4.3)	18 (4.5)

^a^ Multiple choices

**Fig. 2:**
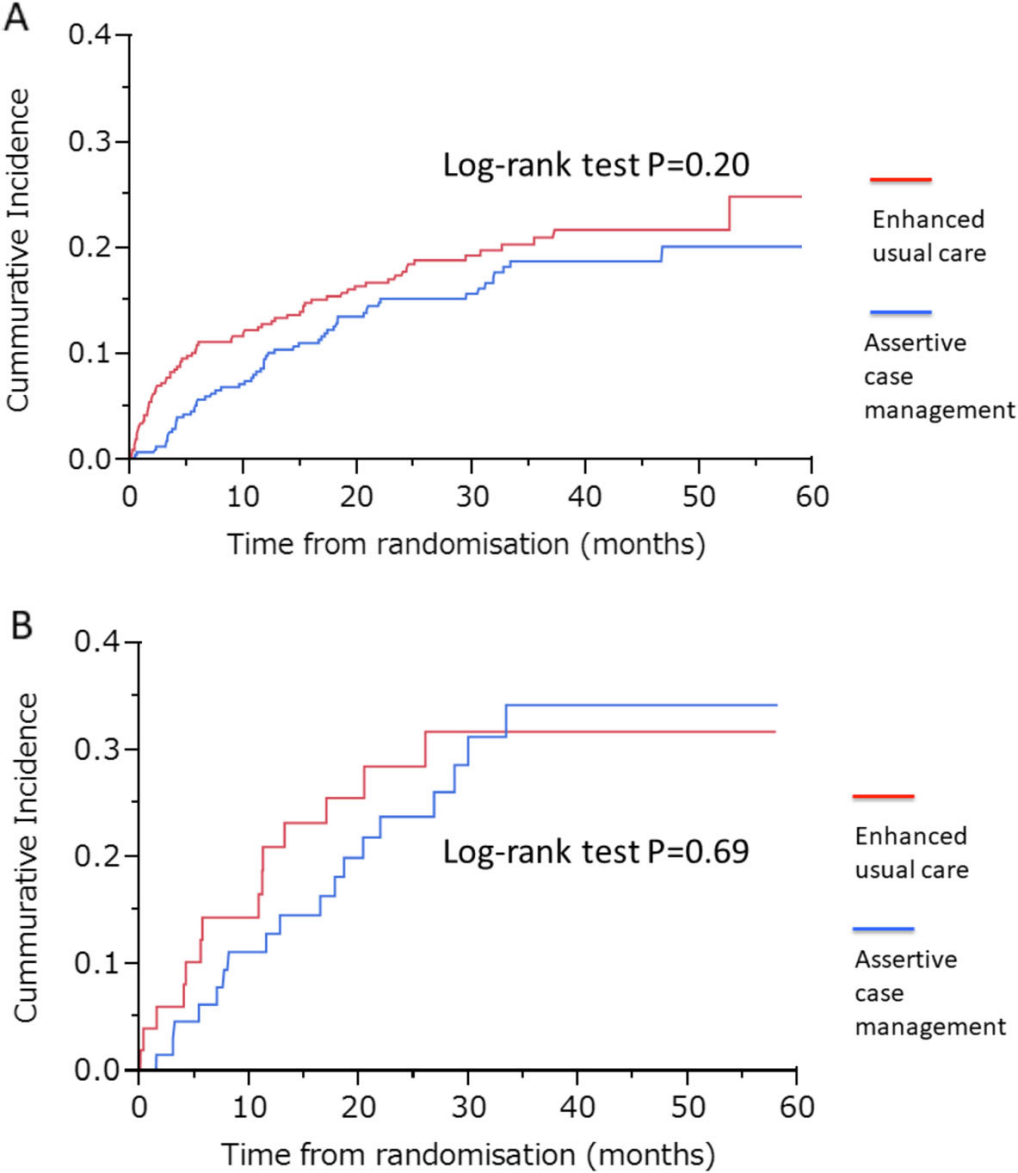
**a** Kaplan-Meier curve for incidence of first episode of recurrent suicidal behaviour in Axis I group (attempted suicide or dying by suicide). **b** Kaplan-Meier curve for incidence of first episode of recurrent suicidal behaviour of the Axis I + II group (attempted suicide or dying by suicide)

Regarding the primary outcome of the incidence of first recurrent suicidal behaviour at 6 months after randomisation, we found significant differences between the Axis I/CM group and the Axis I/EUC group (RR 0.51, 95% CI 0.31 to 0.84) (Table 2). In addition, the differences were unclear, with similar RR between the Axis I + II/CM group and the Axis I + II/EUC group (RR 0.44, 95% CI 0.14 to 1.40) (Table 3). The survival curves for the Axis I + II/CM group and the Axis I/CM group were not clearly different from those of the Axis I + II/EUC group and the Axis I/EUC group (Fig. 2a, b). Regarding the secondary outcome of the first recurrent suicidal attempt at 12 months after randomisation, we observed no clear differences between two groups, but RR values between the Axis I + II/CM group and the Axis I + II/EUC group (RR 0.61, 95%CI 0.26 to 1.42) were similar to those between the Axis I/CM group and the Axis I/EUC group (RR 0.73, 95% CI 0.49 to 1.10) (Tables 2 and 3).

**Table 2:** First recurrent suicidal behaviour (attempted suicide or dying by suicide) of Axis I group. The primary and secondary outcome measures were the incidence proportion of the first episode of recurrent suicidal behaviour (attempted suicide or dying by suicide) at 6 and 12 months after randomisation, respectively

	Primary outcome 6 months (*n* = 734)	Secondary outcome 12 months (*n* = 692)
Intervention vs control	21/354 vs 44/380	35/338 vs 50/354
Unadjusted risk ratio	0.51 (0.31–0.84), *P* = 0.009	0.73 (0.49–1.10), *P* = 0.133
(Imputed missing data) ^*^	0.49 (0.30–0.81), *P* = 0.005	0.72 (0.48–1.08), *P* = 0.112
Adjusted risk ratio ^+^	0.53 (0.32–0.87), *P* = 0.012	0.75 (0.50–1.12), *P* = 0.156
(Imputed missing data) ^§^	0.52 (0.32–0.86), *P* = 0.010 ^*^	0.72 (0.48–1.08), *P* = 0.110

^*^age and sex adjusted

The data included the number of events/population for the intervention participants (assertive case management) or for the control participants (enhanced usual care), or the risk ratio (95% CI). ^*^Risk ratios with data imputed for individuals who missed the assessment. ^+^Risk ratios adjusted by use of regression models for the randomisation factors of sex, age, and history of previous suicide attempts before the current episode. §Risk ratios with data imputed for individuals who missed the assessment and adjusted by use of regression models for the randomisation factors of sex, age, and history of previous suicide attempts before the current episode

**Table 3:** First recurrent suicidal behaviour (attempted suicide or dying by suicide) of Axis I+II group. The primary and secondary outcome measures were the incidence proportion of the first episode of recurrent suicidal behaviour (attempted suicide or dying by suicide) at 6 and 12 months after randomisation, respectively

	Primary outcome 6 months (*n* = 111)	Secondary outcome 12 months (*n* = 104)
Intervention vs control	4/63 vs 10/48	8/59 vs 10/45
Unadjusted risk ratio	0.44 (0.14–1.40), *P* = 0.164	0.61 (0.26–1.42), *P* = 0.252
(Imputed missing data) ^*^	0.44 (0.14–1.41), *P* = 0.167	0.61 (0.26–1.44), *P* = 0.261
Adjusted risk ratio ^+^	0.46 (0.15–1.45), *P* = 0.184	0.66 (0.29–1.48), *P* = 0.310
(Imputed missing data) ^§^	0.45 (0.14–1.43), *P* = 0.174	0.69 (0.30–1.58), *P* = 0.376

^*^age and sex adjusted

The data included the number of events/population for the intervention participants (assertive case management) or for the control participants (enhanced usual care), or the risk ratio (95% CI). ^*^Risk ratios with data imputed for individuals who missed the assessment. ^+^Risk ratios adjusted by use of regression models for the randomisation factors of sex, age, and history of previous suicide attempts before the current episode. ^§^Risk ratios with data imputed for individuals who missed the assessment and adjusted by use of regression models for the randomisation factors of sex, age, and history of previous suicide attempts before the current episode

## Discussion

### Major findings

The present findings revealed that assertive case management was significantly effective in suicide attempters who did not have a comorbid Axis II disorder with an Axis I disorder, and might also be effective in suicide attempters who had a comorbid Axis II disorder with an Axis I disorder.

All of the study participants had a primary diagnosis of an Axis I disorder according to the inclusion criteria. Participants in the ACTION-J trial were encouraged to adhere to psychiatric treatment for their Axis I disorders. Therefore, the current results indicated that a combination of assertive case management and standard psychiatric treatment could reduce the recurrence of suicidal attempts in suicide attempters. Thus, our intervention may be applicable to other settings, such as psychiatric hospitals, and community-based mental health services. However, confirming the effectiveness of the intervention in other settings will require further investigation.

In the current study, RR of the first recurrent suicide attempt within 12 months increased, and this trend was the same as that observed in the intervention group of the ACTION-J trial. In the ACTION-J trial, a case management intervention was delivered less frequently after the 6-month time point. Less frequent intervention might have decreased the effectiveness of the case management intervention. The potential influence of comorbid Axis II disorders on the reduced effectiveness of case management intervention should be considered. Axis II disorders require more intense psychiatric treatment to stabilize the psychiatric conditions involved.

The current results suggest that assertive case management might be an appropriate option for patients with a primary diagnosis of Axis II disorder. However, future trials are required to confirm the effectives of interventions on patients with a main Axis II disorder. A previous randomised controlled trial examined an intervention with a joint crisis plan for the prevention of recurrent suicidal behaviour in BPD patients. Joint crisis plan interventions involve care that is agreed between a patient and their health providers. Unfortunately, the study did not reveal a clear difference in the incidence of recurrence of suicidal behaviour at 6 months between the intervention group and the usual care group [15].

### Strengths and limitations

The current study was a secondary analysis on subgroup comparisons in a randomised controlled trial. We compared an assertive case management intervention with enhanced usual care as a control condition among participants with Axis I and Axis II comorbidity using prospective evaluation of psychiatric diagnosis. The present study involved several limitations that should be considered. First, the number of participants with comorbid Axis II and Axis I disorders was limited. In addition, because our study was a secondary analysis of the trial, it lacked sufficient statistical power for outcomes with few events or small samples. Second, suicide attempters whose primary psychiatric diagnosis was an Axis II disorder were not included in our trial. Third, we were unable to obtain detailed diagnostic data for personality disorders because of the short hospital stays. Fourth, it is important to prevent suicide attempts by adolescents and young people [16]; however, the present study did not include this age group. Finally, unmeasured confounding factors and imperfectly adjusted models may have influenced the results. The analysis model was performed to the best of our knowledge, but it is possible that the selection of variables and their categorization within the models affected our results.

## Conclusions

Assertive case management might have similar effects on suicide-attempting patients with comorbid Axis I and II psychiatric diagnoses to those among patients who attempted suicide with only an Axis I diagnosis. Interventions may be applicable in other settings, such as psychiatric hospitals and community-based mental health services.

## Data Availability

The datasets used and/or analysed during the current study are available from the corresponding author on reasonable request.

## Abbreviations

Axis I group: Subjects who had Axis I diagnosis without Axis II comorbidity
Axis I + II group: Subjects who had comorbid Axis I and II diagnoses
CM group: Case management group
Axis I/CM group: Subjects who had Axis I diagnosis without Axis II comorbidity allocated to assertive and continuous case management
Axis I + II/CM group: Subjects who had comorbid Axis I and II diagnoses allocated to assertive and continuous case management
DSM-IV-TR: The Diagnostic and Statistical Manual of Mental Disorders, Fourth Edition, Text Revision
EUC group: Enhanced usual care group
Axis I/EUC group: Subjects who had Axis I diagnosis without Axis II comorbidity allocated to enhanced usual care
Axis I + II/EUC group: Subjects who had comorbid Axis I and II diagnoses allocated to enhanced usual care
MINI: Mini-International Neuropsychiatric Interview
